# Introducing Step-Down Summaries to the Intensive Psychiatric Care Unit

**DOI:** 10.1192/bjo.2024.92

**Published:** 2024-08-01

**Authors:** Ewan Mahony, Zoe Johnston

**Affiliations:** NHS Lothian, Edinburgh, United Kingdom

## Abstract

**Aims:**

The intensive psychiatric care unit (IPCU) is a 10-bedded unit which houses some of the most unwell psychiatric inpatients, generally those with psychosis and mania who require enhanced care and restriction. Admissions can be long and involve high levels of clinical complexity. This project identified the need for clear communication at the point of discharge with regards to rationale for decision making, mental health act status, risk and outstanding issues. The aim was to develop and test a tool for communicating this: the step-down summary.

**Methods:**

Three plan, do, study, act cycles were run. The first involved creating a draft proforma and testing this with 3 complex patients, gathering qualitative feedback from receiving clinicians. The proforma was then improved and a full-scale trial including all patients with stays of 2 weeks or more was conducted, a total of 18 patients. Data were collated on the timing of summary completion and further improvements to the proforma were made based on consultant feedback. Finally, a third cycle was run to establish whether the new process was sustainable between rotating trainees.

**Results:**

Initial feedback was positive with clinicians highlighting that the summaries saved time reading extensive notes, clearly identified outstanding tasks, and helped with final discharge document writing. It became clear that there was a need to agree a cut-off time of how long a patient should be in IPCU to merit a stepdown summary. Of the 18 patients who met this cut-off in the 2nd cycle all had a stepdown summary at the point of transfer with 89% of these fully complete before their next clinical review. During the 3rd cycle, there were 19 relevant patients only one of whom did not have a summary, due to their transfer coinciding with trainee leave. Feedback remained positive, highlighting that the summaries avoided duplication of work.

**Conclusion:**

Overall, the use of stepdown summaries proved useful to receiving clinicians in both communicating important information and in saving further time when later creating final discharge documents. It was sustainable between trainees, however there remained an issue with these not being produced during trainee leave. It may be useful to consider alternate clinicians who can support with the production of summaries to minimise this as well as measuring more clear clinical outcomes, such as the repetition of investigations. This would support an expansion to other UK IPCUs.

